# Multiple Giant Pilomatricomas of the Shoulder and Upper Limbs

**Published:** 2018-09-28

**Authors:** Rina Hikiami, Natsuko Kakudo, Naoki Morimoto, Masakatsu Hihara, Kenji Kusumoto

**Affiliations:** Department of Plastic and Reconstructive Surgery, Kansai Medical University, Osaka, Japan

**Keywords:** benign skin tumor, calcifying epithelioma, pilomatricoma, giant pilomatricoma, multiple pilomatricomas

## DESCRIPTION

A 35-year-old Japanese man presented with large pedunculated tumor masses on his right shoulder and both upper extremities. We noted 6 tumors, which measured up to 8.0 × 7.0 cm. All 6 tumors were surgically removed. A histopathological examination revealed that all 6 tumors were pilomatricomas.

## QUESTIONS

1. How does pilomatricoma typically present?
2. Which diseases are associated with multiple pilomatricomas?
3. Why do some pilomatricomas become giant?
4. How is giant pilomatricoma distinguished from pilomatrix carcinoma?

## DISCUSSION

Pilomatricoma was first reported in 1880 by Malherbe and Chenantais^1^ as “calcifying epithelioma.” It is a relatively common benign skin tumor originating from hair follicle matrix cells. It typically presents as a slow-growing subcutaneous or intradermal nodule on the face or upper extremities in girls. The long axis of the tumor is typically 0.5 to 3.0 cm, and cases of tumors larger than 5 cm are rare. Although there is no clear definition of “giant,” a tumor with an axis larger than 5 cm is generally considered to be giant. In 1974, Krausen et al^2^ described the first case of giant pilomatricoma measuring 5 × 6 × 7 cm. Giant pilomatricomas are rare and represent a small proportion (<10%) of pilomatricomas.^3^

While the tumor is typically a solitary lesion, multiple pilomatricomas in the same individual have been reported in approximately 2% to 3.5% of cases.^4^ Multiple pilomatricomas have been associated with myotonic dystrophy, Rubinstein-Taybi syndrome, Gardner syndrome, and Turner syndrome. In this case, there was no medical or family history of skin disease or similar tumors.

In a search of the literature on pilomatricoma, only 10 cases of multiple giant pilomatricomas with an axis larger than 5 cm, including this case, were extracted, indicating that multiple giant pilomatricomas are extremely rare. There were 6 male and 4 female patients. Their ages ranged between 13 and 63 years (mean ± SD = 31.1 ± 14.3). Patients with multiple giant pilomatricomas were slightly older. Some reasons have been proposed for these tumors becoming giant: (1) an external stimulation such as scratching or clothing; (2) inflammation around the tumor caused by bacterial infection, and cytokines such as growth factors being released^5^; (3) in the case of obesity, thick subcutaneous fat prevents the destruction of the tumor. In our case, although there was no obvious inflammation, tumors occurred on the right shoulder and upper arms, and the patient had a body mass index of 33. Therefore, the tumors in the present case appear to have become enlarged because of a combination of these reasons.

Giant pilomatricoma needs to be distinguished from pilomatrix carcinoma. The locally aggressive malignant equivalent of pilomatricoma, which was first identified by Lopansri and Mihm^6^ in 1980, has been referred to as “pilomatrix carcinoma,” “malignant pilomatricoma,” “trichomatrical carcinoma,” or “calcifying epitheliocarcinoma of Malherbe.” Pilomatrix carcinoma occurs more often in middle-aged to elderly individuals, and the average size of the tumor is slightly larger than that of benign pilomatricoma.^7^ The histological features of pilomatrix carcinoma include the proliferation of hyperchromatic and vesicular basaloid cells with numerous mitoses and infiltration into fat or the underlying structures.^7^ Several cases of lymph node metastases have been reported to date, while systemic (mainly pulmonary) metastases developed in several patients.^8^

There were no malignant findings of any lesions in a histological examination in the present case, and tumors did not differ from pilomatrix carcinoma but were large. There have been no signs of recurrence at the excised sites or newly growing tumors at other sites 1 year after surgery. Careful long-term follow-up observations will be necessary in the future.

## Figures and Tables

**Figure 1:**
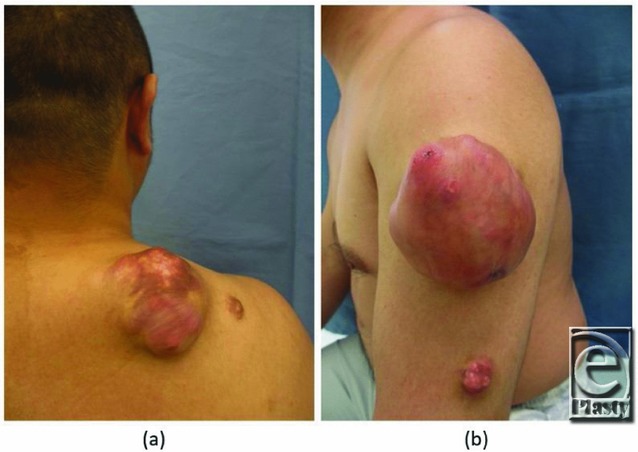
Preoperative findings. (a) 8.0 × 7.0-cm tumor and 1.5 × 1.5-cm tumor on the right shoulder. (b) 8.0 × 6.0-cm tumor and 3.0 × 2.0-cm tumor on the left upper arm.

**Figure 2:**
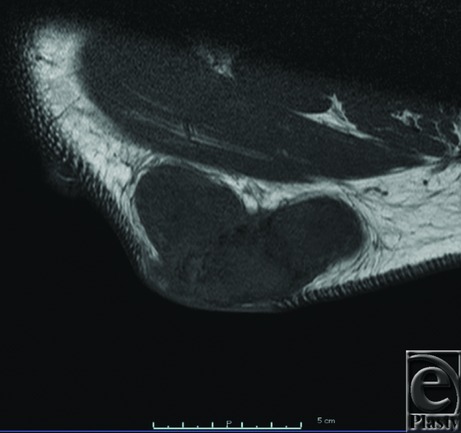
Signal intensity of the tumor on the right shoulder on magnetic resonance images. On this T1-weighted image, the tumor shows iso/low signal intensity.

**Figure 3:**
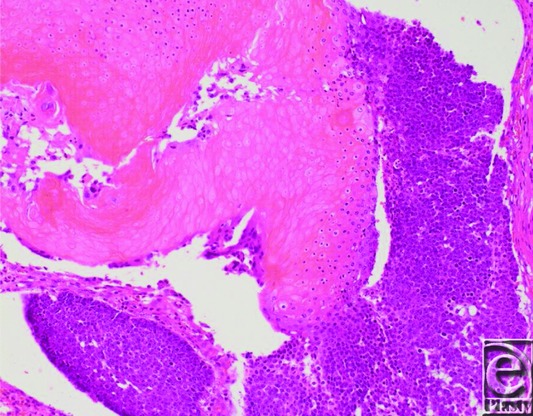
Histological findings of the excised tumor. Hematoxylin and eosin staining shows a dermal tumor mainly composed of shadow cells. The black arrow indicates shadow cells. Original magnification ×100.

**Figure 4:**
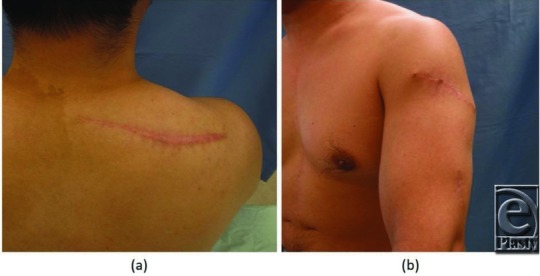
Eight months postoperatively, no recurrent signs were visible at the operative site. (a) The right shoulder. (b) The left upper arm.
